# Implementation of Hand Gesture Recognition Device Applicable to Smart Watch Based on Flexible Epidermal Tactile Sensor Array

**DOI:** 10.3390/mi10100692

**Published:** 2019-10-12

**Authors:** Sung-Woo Byun, Seok-Pil Lee

**Affiliations:** 1 Department of Computer Science, Graduate School, SangMyung University, 20, Hongjimun 2-gil, Jongno-gu, Seoul 03016, Korea; 123234566@naver.com; 2 Department of Electronic Engineering, SangMyung University, 20, Hongjimun 2-gil, Jongno-gu, Seoul 03016, Korea

**Keywords:** gesture recognition, flexible epidermal tactile sensor array, wearable device, wearable sensors

## Abstract

Ever since the development of digital devices, the recognition of human gestures has played an important role in many Human-Computer interface applications. Various wearable devices have been developed, and inertial sensors, magnetic sensors, gyro sensors, electromyography, force-sensitive resistors, and other types of sensors have been used to identify gestures. However, there are different drawbacks for each sensor, which affect the detection of gestures. In this paper, we present a new gesture recognition method using a Flexible Epidermal Tactile Sensor based on strain gauges to sense deformation. Such deformations are transduced to electric signals. By measuring the electric signals, the sensor can estimate the degree of deformation, including compression, tension, and twist, caused by movements of the wrist. The proposed sensor array was demonstrated to be capable of analyzing the eight motions of the wrist, and showed robustness, stability, and repeatability throughout a range of experiments aimed at testing the sensor array. We compared the performance of the prototype device with those of previous studies, under the same experimental conditions. The result shows our recognition method significantly outperformed existing methods.

## 1. Introduction

Ever since the development of digital devices, the recognition of human gestures has played an important role in many Human-Computer interface (HCI) applications, permitting interaction in a natural and comfortable way [1,2,3,4]. Hand gesture recognition has the advantage of being applicable to a range of applications, such as handling presentations, controlling drones, and more [5]. A universal remote-control system using hand gestures is presented in [6]. Hand gesture recognition is achieved using two main kinds of sensors: contact sensors and non-contact sensors. The non-contact methods are primarily based on visual technologies such as camera sensors, Kinect, and Leap Motion controller (LMC), which do not require attaching the sensors to the human body, as reported by various studies [7,8,9,10,11,12,13]. Contact methods identify gestures by analyzing the signal acquired from contact sensors, which are wrapped around the user’s arm or wrist, or are attached to a glove that the user wears [14,15,16]. They have a wider recognition range than the non-contact methods, without constraints such as limited range and the sight of sensors, and relatively accurate information can be acquired due to the direct contact with the user. For this reason, various wearable devices have been developed, and inertial sensor, magnetic sensor, gyro sensor, electromyography (EMG), force-sensitive resistors (FSRs), and others have been used to identify gestures. In particular, the EMG sensor has been used in many studies on gesture recognition [17,18,19]. Many researchers used EMG sensors for recognizing the intention of an operator [17,20,21,22]. Recently, to control digital devices, Thalmic Labs Co. developed an EMG-based gesture recognition device, which is referred to as Myo [5]. The device was designed as an armband bracelet to measure EMG signals from the forearm muscles. EMG-based methods have become more important in the practical application of surface electromyography [22]. The main challenges of EMG-based methods are the weak signal intensity with noise [23]. Generally, the amplitude range is 0–105 mV and the bandwidth is 0.5–2 kHz, so it is easily interfered in by the external noise and the acquisition device itself [22,24].

Another gesture recognition approach is the use of FSRs. FSRs sensors detect muscle activity by measuring and monitoring changes in resistance generated by movements of the muscles [25,26]. Since the muscle contraction occurs the longitudinal elongation and the expansion of its cross-sectional area, it is possible to detect the muscular activity by monitoring the swelling of muscles by FSRs sensor [24]. FSRs sensor is robust to noise compared to the other bio signal measurements, but the output voltage of FSRs sensors is nonlinear due to relationship between output voltage and resistance [25]. In addition, since FSRs sensor is a thin film, and thus an input device with FSRs sensor should become rigid, which causes discomfort in wearing [24].

Mechanomyography (MMG) can also be used to detect muscular activities. Muscular activity is identified by mechanical vibration, which is generated by the tremor of each muscle fiber [24]. MMG-based methods commonly use an accelerometer [27,28] and a microphone [29,30]. However, MMG based on an accelerometer can only be used when the magnitude of acceleration is distinguishable compared to acceleration due to gravity and motion. MMG based on a sound transducer is reliable only in a silent space [24].

These sensors have been shown to be successful in many studies over the past two decades. However, there are still different drawbacks for each sensor, which affect the detection of gestures. To overcome these problems and accurately recognize gestures, we developed a novel gesture recognition method using a Flexible Epidermal Tactile Sensor Array (FETSA) based on strain gauges to sense deformations. Such deformations are transduced to electric signals. By measuring the electric signals, the sensor array can estimate the degree of deformations, including compression, tension, and twist caused by movements of the wrist. The principle of FETSA is similar to that of MMG sensors and FSR sensors, but its flexibility provides enhanced usability in terms of wearing the sensor. The sensor guarantees linearity, in contrast with FSRs sensors. To test the performance of the sensor, we fabricated a prototype clip-type device, and conducted comparison tests using the porotype device. We compared the sensor with a commercial EMG sensor and an FSRs sensor, which are commonly used in gesture recognition studies. Furthermore, we compared the porotype device with previous studies, under the same experimental conditions. We conducted additional experiments using gestures defined in this research. The resulting recognition method significantly outperformed existing methods.

## 2. Principle of Flexible Epidermal Tactile Sensor Array

**Figure 1:**
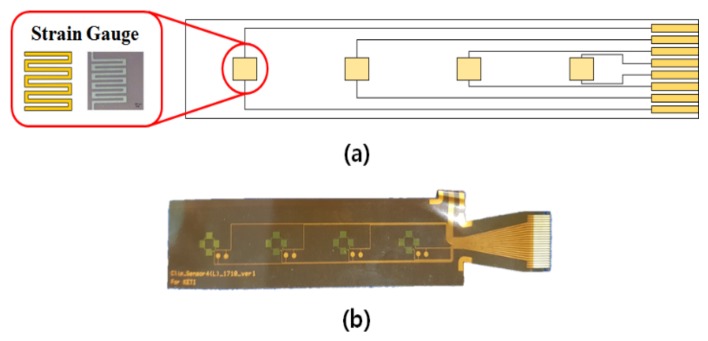
Flexible Epidermal Tactile Sensor Array (FETSA). (**a**) Design of the FETSA. (**b**) Fabricated sensor.

When a gesture occurs, the length and thickness of the muscles around the wrist change during concentric contraction and eccentric contraction, changes which are classified as dynamic contraction. For concentric contraction, related muscles shorten and thicken while muscular force is generated. In eccentric contraction the muscles involved lengthen and become thinner. Isometric contraction corresponds to static contraction; there is no change in the muscle length, although the muscles generate force. Isometric contraction occurs when maintaining a posture or holding an object. Therefore, in order to detect hand movements when gestures occur, we must measure the changes in muscles, whether concentric contraction or eccentric contraction. However, because the EMG sensor measures all three types of contractions, devices based on the EMG sensor require additional signal processing to distinguish isometric contraction from the other two contractions of interest. To unambiguously detect concentric contraction and eccentric contraction, we developed a Flexible Epidermal Tactile Sensor Array to measure the movement of muscles in a reliable and convenient way. The proposed sensor is shown in Figure 1a.

In sensor design, the number and location of sensors are important in order to recognize gestures. The initial model was fabricated with 16-channel sensors, so that it could wrap around the whole of a wrist [31]. Based on preliminary experiments, the final model has four sensors. To detect the movement of wrist muscles, sensors are positioned over the muscles responsible for wrist movements. The device was designed using flexible polyimide, so that it could be worn on the wrist to improve its fit to the user’s body surface. Strain gauges are located on the flexible substrate. Figure 1b shows the fabricated sensor array. Depending on the movement of the wrist, the analog resistance value of the flexible array sensor is processed using a circuit and converted into a digital value. This value is then used for gesture recognition.

**Figure 2:**
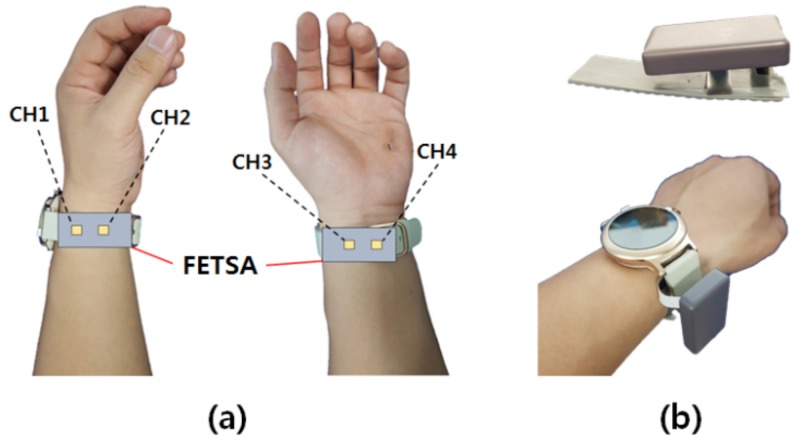
(**a**) Location of sensors. (**b**) How to wear the device.

Figure 2b shows the gesture recognition device based on FETSA. It was designed as a clip so that it could be worn with a smart watch. The data acquisition board includes a serial communication unit, through which the sensor signal is recorded. The baud rate is set to 115200 for real-time processing. Sixty data units per second are acquired through the device. The four sensors of the device detect the activities of muscles responsible for the movement of the wrist as shown in Figure 2a. Channel 1 is located on the abductor pollicis longus muscle, which deals with the up and down movement of the thumb. A sensor is located on each of the muscles responsible for the movement of the wrist.

**Figure 3:**
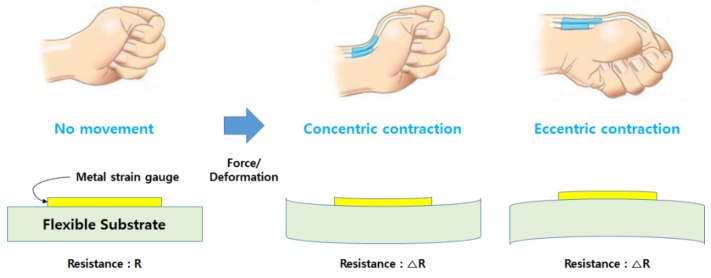
Change of a strain gauge and resistance caused by the movement of a wrist.

As discussed above, concentric and eccentric contraction in muscles under the wrist occur when people make hand gestures. When the fist is twisted down, as shown in Figure 3, eccentric contraction occurs at the extensor pollicis brevis muscle. This contraction influences the strain gauge of the sensor located on the muscle. Force generated from the muscle is transmitted to the sensor, raising the resistance value due to the expansion of the sensor. In contrast, when the fist is twisted up, concentric contraction occurs in the muscle, decreasing the resistance value of the sensor. The proposed sensor detects the activities of muscles under the wrist by measuring these deformations of the sensor to detect gestures.

## 3. Gesture Recognition with FETSA

**Figure 4:**
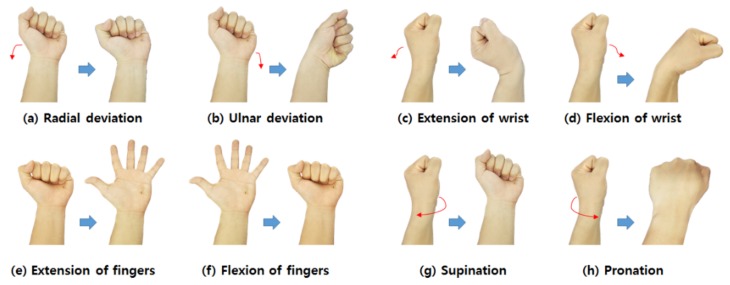
Eight motions of the wrist and fingers: (**a**,**b**) radial and ulnar deviation of the wrist; (**c**,**d**) extension and flexion of the wrist; (**e**,**f**) extension and flexion of fingers; (**g**,**h**) supination and pronation of the wrist.

In this section, we explain how the device recognizes gestures. First, we investigated the changes in the signals from each of the sensors according to the movement of the wrist, since the muscles may influence the deformation of adjacent sensors simultaneously. There are eight motions which can be made with wrist and fingers (Figure 4).

Since each muscle is theoretically concerned with different motions, each sensor of the device detects different signals according to the motions. We investigated the changes in the signals when a subject made different motions. The subjects started with a light motion by relaxing the hands before making the eight motions shown in Figure 4.

**Figure 5:**
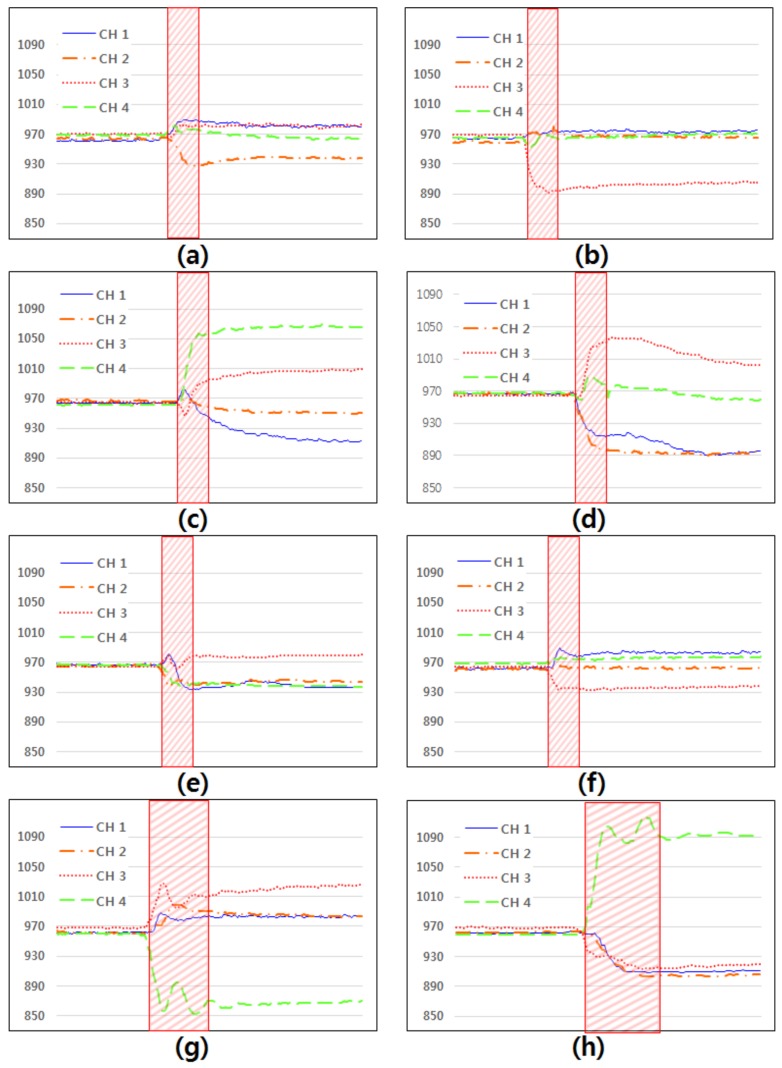
Changes in each of the signals (**a**–**h**) produced during the eight motions shown in Figure 4. The shaded areas indicate when each movement was made.

Figure 5 shows the change in each signal when the eight motions shown in Figure 4 occurred. The changes in the signals acquired from the four channels were different in each motion. For instance, in motion (a) and motion (b), the signals acquired from channel 4 and channel 1 are similar, but the signals are different in channel 2 and channel 3 (Figure 5a,b). The proposed method can distinguish the eight motions using the differences in signals, an observation which verifies that the hand gestures can be recognized.

**Figure 6:**
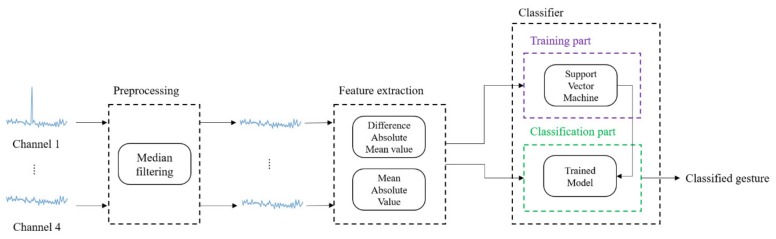
Overview of the process of gesture recognition method using the proposed device.

The entire process of gesture recognition using the proposed device is shown in Figure 6. The process consists of preprocessing, feature extraction, and classification. The steps are explained in detail as follows.

### 3.1. Preprocessing

**Figure 7:**
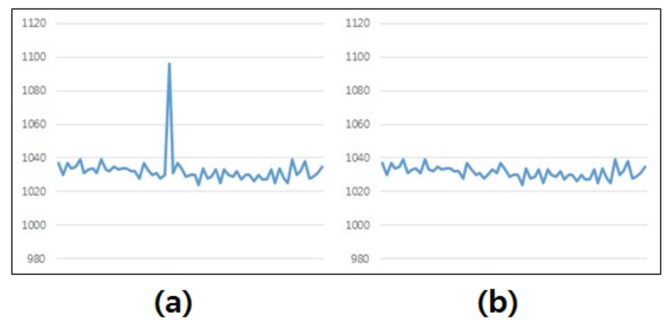
(**a**) Signal noise caused by heartbeat. (**b**) Results after the preprocessing.

While recording bio-signals, mixed signals sometimes occur due to the presence of noise. For example, noise can be recorded from the heartbeat reflected in the artery under the wrist, and interpreted as a movement of the wrist. Such noise leads to the degradation of the quality of the signal, and must therefore be removed. As apparent in Figure 7a, a recording of a stable signal is periodically deformed by heartbeats. As this deformation can cause reduction in the accuracy of gesture recognition, we used a median filter to remove this noise. This approach is effective in removing impulse noises while preserving the important properties of the signal. Figure 7b shows the results after the noise is removed.

### 3.2. Feature Extraction

Since signals acquired from the sensors differ according to the motion, as shown in Figure 5, we extract uncomplicated time series features to distinguish between gestures. Based on the results of the investigation, we selected two features which reflect the change and power of the signal. 

The difference absolute mean value (DAMV) feature vector measures signal change equal to the average absolute difference of two sequential values as follows:

(1) $$DAMV=\frac{\sum_{i=1}^{N-1}\left|X(i)-X((i+1))\right|}{N-1}$$

The mean absolute value (MAV) is a measure of signal power which is equal to the average absolute value of the signal as follows:

(2) $$MAV=\frac{\sum_{i=1}^{N}\left|X(i)\right|}{N}$$

### 3.3. Classification

We used an algorithm based on support vector machines (SVMs), which are well known to be the algorithm with the best generalization among machine algorithms. SVM is a supervised learning model widely used in classification and regression analysis. SVM maps features onto higher dimensions using a kernel function, and distinguishes them according to class, using hyperplanes. An appropriate SVM kernel must be selected to determine the decision boundaries between the different classes. We selected a radial basis function (RBF) kernel for non-linear classification [32].

(3) $$k(x_{i},x_{j})=\exp(-\gamma {\left\|x_{i}-\left.x_{j}\right\|\right.}^{2}),{}^{}\gamma >0$$

Here, *γ* is a kernel parameter which indicates the influence of squared Euclidean distance. We used the LIBSVM library, one of the most-used SVM libraries [32]. The two features mentioned in Section 4.2 were used as input. A classifier classifies inputs using a trained model generated by the training process.

**Figure 8:**
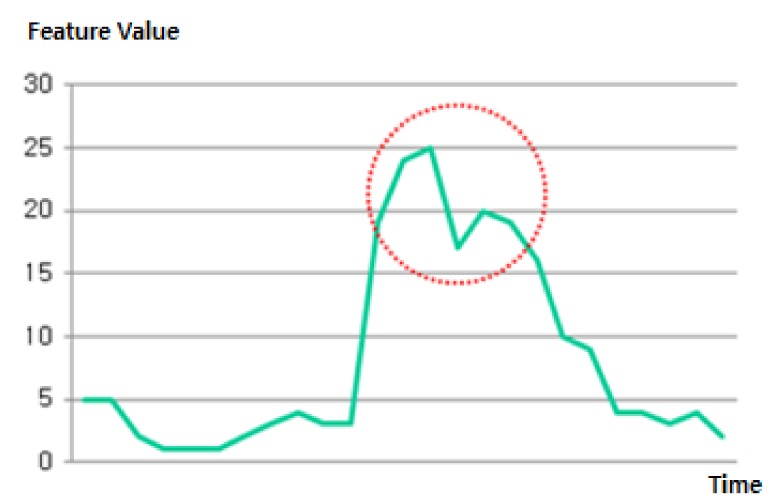
Example of extracted DAMV features. The circled area indicates when the subject made a gesture.

In training, the variation of the signal is high when making a gesture, so it is presumed that the gesture is changed when the DAMV feature falls within the red circle, as shown in Figure 8. An SVM was trained using the MAV and DAMV features by monitoring the changes of signals. In each experiment, we acquired training data from each subject making each gesture for five seconds before the experiments began. To train the machine, we used five seconds of training data for each gesture from each subject. To investigate the optimal kernel parameter, we used several different pairs (C, γ) when the machine was trained, and selected an optimal parameter set empirically. In practice, there are limitations of a training process such as this in training the machine, but it guarantees certain training for specific subjects.

Since the period of feature extraction was about 30 Hz, the classifier was run using the trained model in real-time at 30 Hz. We asked subjects to make a gesture and produced a classification by confirming the concurrence of a classification result and the gesture the subject made.

## 4. Experiments

In order to verify the performance of our proposed method, we conducted a comparison test between the proposed sensor, a commercial EMG sensor, and an FSRs sensor. We compared the accuracy of the proposed method with a commercial gesture recognition device and the results of previous research. To produce an objective comparison, we used the same experimental conditions, including the number of repetitions, gestures, and other factors, as used in previous studies. Lastly, we conducted an experiment using the gestures described above. 

### 4.1. Comparison with EMG Sensor

The FETSA sensor was compared with an EMG sensor, which is the most intuitive and widely-used method for measuring muscle activity. A certified commercial EMG sensor, MyoWare Muscle Sensor of Advancer Technologies, was used. The sensor was attached above the extensor pollicis brevis muscle, which is responsible for the movement of the thumb, to detect the activities of the muscle. The FETSA sensor corresponds with channel 2 in Figure 4a. The sensor signals were recorded when the subject remained motionless and when the subject produced a “thumbs-up” motion. 

**Figure 9:**
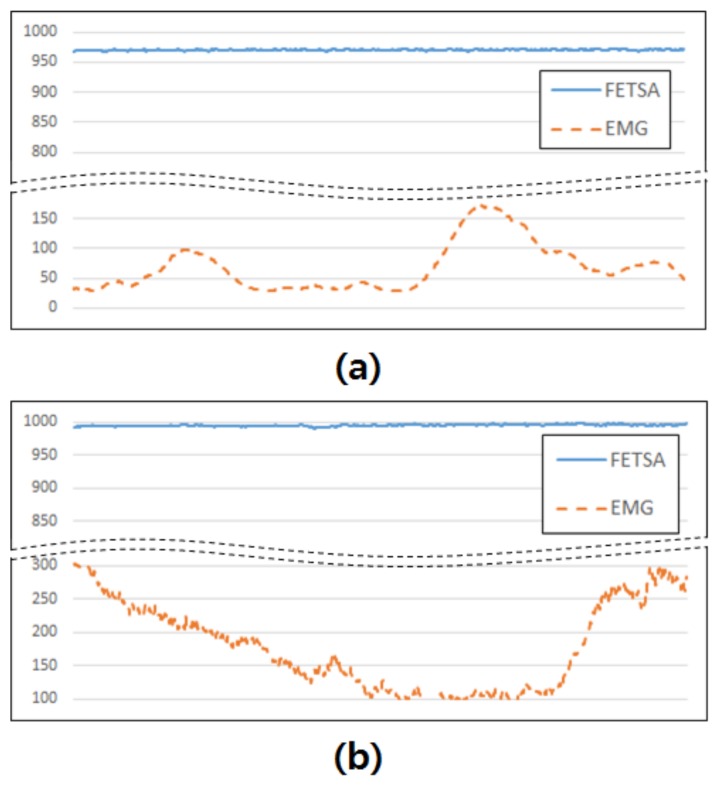
Comparison of the results using FETSA and EMG. (**a**) When the subject remained motionless. (**b**) When the subject produced the “thumbs-up” motion.

Figure 9 shows the results of the comparison. In Figure 9a, signals were acquired when the subject remained motionless with the sensors attached, and in Figure 9b, signals were acquired while the subject produced the “thumbs-up” motion. As shown in Figure 9, the signal acquired from the FETSA was relatively uniform when the subject took no motion or made the “thumbs-up” motion. In contrast, in the EMG sensor, the signal had greater fluidity, even when the subject made no motion (Figure 9a). When the subject made the “thumbs-up” motion, the fluctuation in the signal was large, as it was affected by noise, and also static and dynamic contraction (Figure 9b). To analyze these results, the average and standard deviation of the signals were calculated. When the subjects made no motion, the average of the signal from FETSA was 970.08 and the standard deviation was 0.87, while the average of the signal from EMG was 68.90, with a standard deviation of 39.8. The average of the signal from FETSA was 995.18, and the standard deviation was 1.56. In contrast, the average of the signal from EMG was 67.47, and the standard deviation was 67.46. These results indicate that the FETSA sensor is more robust to noise and more stable than the EMG sensor.

### 4.2. Comparison with the FSR Sensor

Although the robustness against noise of the FETSA sensor was demonstrated in Section 4.1 in comparison with the EMG sensor, it remained to be investigated whether FETSA can recognize gestures effectively. Therefore, we compared FETSA with the FSRs sensor, one of the most widely-used sensors for gesture recognition. A commercial FSRs sensor, RA18-DIY of Marveldex, was used. We attached the sensor over the same muscle that was used for the EMG and FETSA sensors in the previous section. 

**Figure 10:**
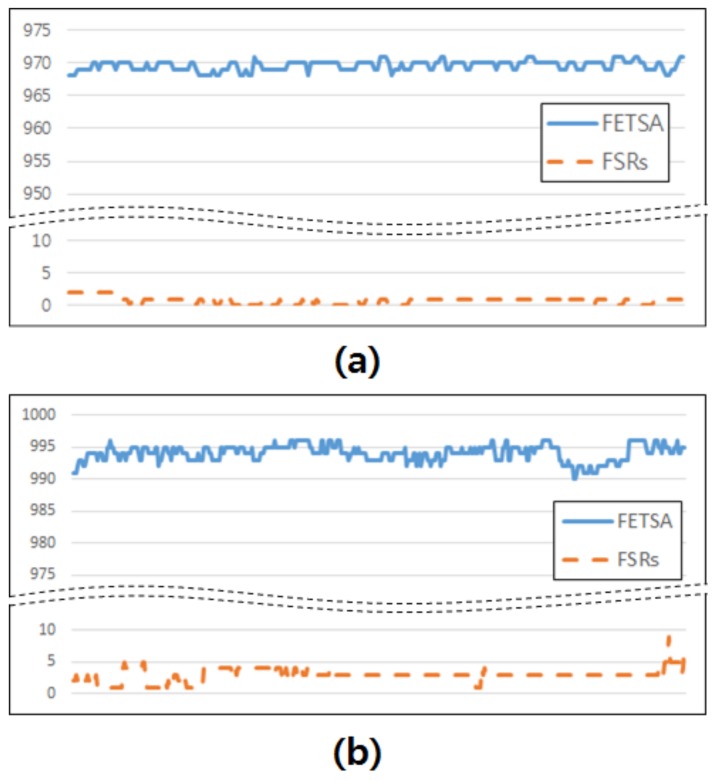
Comparison of the results using FETSA and FSRs: (**a**) the subject remained motionless, (**b**) the subjects made the “thumbs-up” motion.

As shown in Figure 10, since the FSRs sensor is robust to electric noise, it acquired a more stable signal than the EMG sensor. When the subject was motionless, the average of the signal from the FETSA was 969.57, and standard deviation was 0.76. In case of FSRs, the average and standard deviation of the signal were 0.74 and 0.6 respectively. When the subject made the “thumbs-up” motion, the average of the signal from FETSA was 994.05, and the standard deviation was 1.26. In the case of the FSRs, the average and standard deviation of the signal were 2.98 and 1.01 respectively. Comparing both (a) and (b), the standard deviation of signals acquired from FETSA and FSRs sensors were similar, with a small fluctuation of 0.1–0.2. However, the sensors showed a difference in mean difference values. In the case of FETSA, the mean difference value was 26.48 between when the subject made the “thumbs-up” motion and when the subject remained motionless. In the FSRs, the mean difference value was 2.24. It is difficult to distinguish between the two conditions based on the signal from the FSR sensor, because the mean difference is low, with high standard deviation. However, it is easier to differentiate between the two conditions from the FETSA signals, which have a larger mean difference. The reason why the FSRs sensor does not have a high mean difference is that the signal does not increase when raising the thumb, due to the non-linearity of the FSRs sensor. Overall, these results indicate that the FETSA sensor is more effective than the FSRs sensor.

### 4.3. Repeatability

Good repeatability is crucial for sensors, so we conducted an experiment to verify the repeatability of FETSA. A subject wearing the device was asked to clench and open his fist 10 times in a row.

**Figure 11:**
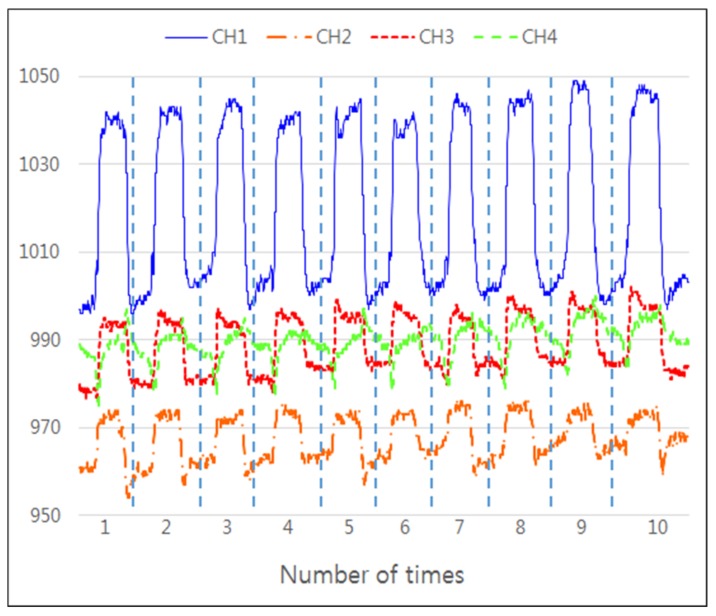
Results of repeatability tests.

Every channel of the sensor array was used in the repeatability test, and the results are shown in Figure 11. The same signal pattern was observed for each trial. To quantitative the results, the peak values of signals from the four channels were measured, and their averages and standard deviations were calculated. The average of the peak value was 1040.27 in channel 1, and the standard deviation was 0.93. The average of all channels was 998.94 and the standard deviation was 0.81. The standard deviations were very small compared to the average of peak values, indicating FETSA’s ability to accurately measure repeated muscle activity.

### 4.4. Comparison with Contact Gesture Recognition Study

Pyeong-Gook Jung et al. [24] introduced a new method to detect muscular activity using air-pressure sensors. This approach overcomes the drawbacks of EMG and MMG sensors in detecting muscle activity and recognition of hand gestures. These researchers detected muscular activity by measuring the change in air pressure at air-pressure sensors contacted with the muscle of interest. They used fuzzy logic to determine gestures from the role of the muscles in each gesture.

**Figure 12:**
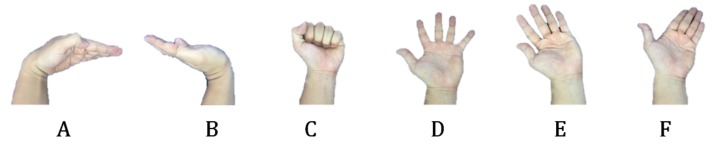
The six gestures that were defined in Jung’s research.

To compare the performance of FETSA with that of the previous study, we used the six gestures defined in Jung’s research (Figure 12). The test conditions were made as similar as possible, to ensure valid comparisons (Table 1).

The average accuracy of FETSA was 99.1% while the average accuracy of the previous study was 95.35% [24]. FETSA was therefore more effective at determining the same gestures than the previous study.

### 4.5. Comparison with a Commercial Gesture Recognition Device

**Figure 13:**
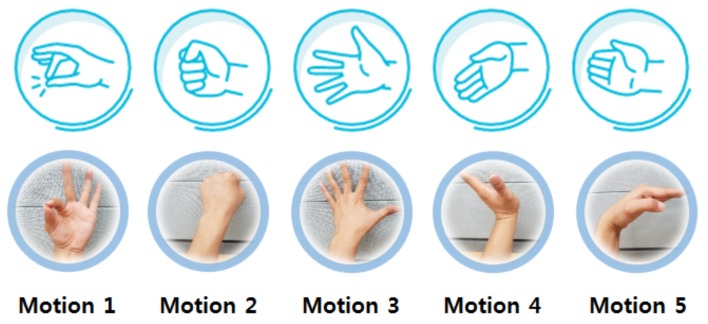
The five gestures provided by Myo.

Myo, which is developed by Thalmic Labs Co., is a commercial gesture recognition device [5]. It measures EMG signals from sensors worn on the user’s arm to control other digital devices. Myo provides five gestures (Figure 13).

We asked subjects to make the gestures while wearing the proposed device and Myo, and compared the accuracy of gesture recognition from the two devices. The subjects participating in the test were four men and four women. The results are presented in Table 2.

The average error rate of Myo was 17.5%. In contrast, the average error rate of FETSA was 4.25%. Myo recognized motion 3 as motion 2, and failed to recognize motion 1, resulting in a considerable increase in the average error rate. However, FETSA had a low average error rate and recognized the five gestures more accurately than Myo. 

### 4.6. Hand Gesture Recognition with an FETSA Sensor

**Figure 14:**
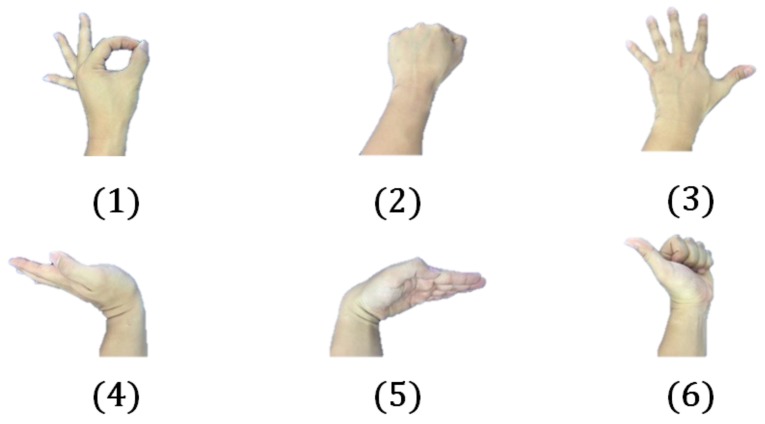
The six gestures defined in this research.

We performed a recognition experiment using the gestures defined in this research. The six hand gestures are shown in Figure 14: pinch of the finger ((1) in the Figure); flexion and extension of the fingers ((2) and (3)); flexion and extension of the wrist ((4) and (5)); extension of a thumb from a fist (6).

The eight subjects participating in this experiment were six men and two women. Before the experiment began, data from the subjects was used to train the SVM to recognize the gestures for five seconds. Each experiment was conducted 30 times per gesture, and the researcher randomly selected each gesture. The subjects made a gesture according to the researcher’s instructions. The gesture recognition tests were repeated 1440 times. The results for the eight subjects are shown in Table 3. The average success rate for gesture recognition was 97.8%, and the number of misclassifications was very low at 2.2%.

## 5. Discussion and Conclusions

In this study, we developed a new gesture recognition method using FETSA, based on strain gauges to sense deformations. The sensor array was designed to overcome the drawbacks of other sensors and accurately recognize gestures. We fabricated a prototype clip-type device, providing enhanced usability in terms of wearing the sensor. Preprocessing algorithms were developed to remove noise from the acquired electrical signals. DAMV and MAV features were extracted from the signals, and gestures were recognized by an SVM, using the extracted features. The sensor array was shown to be able to analyze the eight motions of the wrist. We compared the performance of the sensor with those of a commercial EMG sensor and an FSRs sensor, which are commonly used in gesture recognition studies, under the same experimental conditions. We conducted additional experiments using the gestures defined in this research. As seen in the results, the proposed recognition method performed extremely well when compared with existing methods. However, it is difficult to directly compare our results with those of many other studies, due to the very different conditions involved, such as different types of gestures and different numbers of gestures.

Table 4 shows the results of previous gesture recognition studies which used a wide variety of techniques. Most recognition systems obtained accuracies of 80–90%, with an average accuracy of 90.93%. The results of this study, which produced 97–99% accuracy over the three experiments indicate that the proposed device is superior to those used in previous studies (Table 2). In future research we plan to study methods using both the movement and the location of a hand, combining the FETSA sensor with the IMU sensor.

## Figures and Tables

**Table 1:** Comparison results for each subject.

Gesture	A	B	C	D	E	F
	Success(Proposed)/Success(previous)/Trial					
Subject A	**30**/30/30	**30**/30/30	**30**/29/30	**30**/29/30	**30**/28/30	**30**/30/30
Subject B	**18**/18/18	**19**/19/20	**22**/21/22	**16**/15/16	**20**/18/20	**15**/15/15
Subject C	**18**/17/18	**15**/14/15	**15**/15/15	**15**/14/15	**15**/15/15	**15**/16/17
Subject D	**20**/19/20	**14**/13/14	**16**/16/16	**18**/16/18	**20**/18/20	**15**/14/15
Subject E	**17**/16/17	**15**/16/17	**18**/17/18	**20**/18/20	**15**/14/15	**15**/14/15
Subject F	**18**/18/18	**15**/15/15	**16**/16/16	**16**/15/16	**17**/16/18	**20**/18/20
Total (%)	**100**/97.5/100	**97.3**/96.4/100	**100**/97.4/100	**100**/93.0/100	**99.1**/92.3/100	**98.2**/95.5/100

**Table 2:** Results of comparison tests between the proposed device and myo.

Gesture	Myo (Error Rate)	Proposed Device (Error Rate)
Motion 1	22.5	2.5
Motion 2	6.25	5
Motion 3	33.75	5
Motion 4	15	5
Motion 5	10	3.75
Total (%)	**17.5**	**4.25**

**Table 3:** Comparison results for all subjects.

Gesture	1	2	3	4	5	6	Total (%)
1	234	0	0	2	0	2	98.3
2	0	235	1	0	3	2	97.5
3	0	0	239	1	1	0	99.2
4	1	2	0	234	1	1	97.9
5	0	3	0	2	232	0	97.9
6	5	0	0	1	3	235	96.3
Total (%)	97.5	97.9	99.5	97.5	96.6	97.9	**97.8**

**Table 4:** Results from previous studies on gesture recognition studies.

Sensor	Application	Algorithm	Accuracy
EMG & FSR [4]	Wrist	SVM	96%
EMG [33]	Finger	LDA	92%
Gyro sensor [1]	Hand, finger	-	98%
infrared sensor [34]	Wrist	Otsu’s threshold	99%
OMTS [35]	Wrist	SVM	93%
EMG+IMU [36]	Wrist	LDA	96%
EMG+Inertial sensor [15]	Wrist	HMM	97.8%
EIT [37]	Wrist	SVM	90%
gyro sensor [38]	Wrist	-	96%
FSR [26]	Wrist	SVM	80%
EMG [2]	Wrist	HMM	89.60%
EMG [39]	Leg	LDA	90%
Flexible msg [40]	Glove	K-NN	93%
Gyro [41]	Hand	HMM	89%
EMG [18]	Brachial muscle	Fuzzy	92%
EMG [20]	Hand, Finger	HMM	90.5%
EMG [42]	Wrist	SVM	86%
MMG [43]	Forearm	LDA	89%
EMG [44]	Forearm, Finger	SVM	83%
EMG [45]	Finger	LDA	90%
MMG [28]	Brachial muscle	QDA	79.66%
